# The potentially protective effect of lithium on the risk of osteoporosis: A nationwide study of 22,912 patients with bipolar disorder

**DOI:** 10.1192/j.eurpsy.2022.431

**Published:** 2022-09-01

**Authors:** S.D. Østergaard, O. Köhler-Forsberg, C. Rohde, A. Nierenberg

**Affiliations:** Aarhus University Hospital - Psychiatry, Department Of Affective Disorders, Aarhus N, Denmark

**Keywords:** Osteoporosis, bipolar disorder, Lithium

## Abstract

**Introduction:**

Osteoporosis, a systemic skeletal disorder associated with substantial morbidity and mortality, has been suggested to be particularly common among individuals with bipolar disorder. Lithium, a mood-stabilizer used as first-line treatment for bipolar disorder, may have bone-protecting properties.

**Objectives:**

We aimed to subject both of these hypotheses to further examination in a nationwide register-based study.

**Methods:**

We compared the incidence of osteoporosis, identified via hospital discharge diagnoses and prescribed medications, between all individuals diagnosed with bipolar disorder and age- and sex-matched controls from the general population (earliest start of follow-up at the age of 40 years) using Cox regression. Subsequently, we followed the patients with bipolar disorder and identified all prescriptions for mood-stabilizing medications. Using Cox regression, we compared the incidence of osteoporosis for patients using lithium, antipsychotics or anticonvulsants, respectively, with that of patients not using these medications.

**Results:**

We followed 22,912 patients with bipolar disorder (median age 50.4 years, 43.4% men) and 114,560 matched controls for 1,215,698 person-years. The incidence of osteoporosis per 1,000 person-years was 8.70 (95%CI:8.28-9.14) among patients with bipolar disorder and 7.84 (95%CI:7.67-8.01) among controls, resulting in a hazard rate ratio (HRR) of 1.15 (95%CI:1.09-1.21). Lithium treatment was associated with reduced risk of osteoporosis (HRR=0.62; 95%CI:0.53-0.72) in a treatment-duration-response-like manner. Treatment with antipsychotics and anticonvulsants was not associated with reduced risk of osteoporosis.

**Conclusions:**

This is the first longitudinal study to show that the risk of osteoporosis is elevated among patients with bipolar disorder, and that treatment with lithium is associated with reduced risk of osteoporosis.

**Disclosure:**

Dr. Østergaard has received the 2020 Lundbeck Foundation Young Investigator Prize. Furthermore, SDØ owns units of mutual funds with stock tickers DKIGI, DKIDKIX, MAJGRO, NBIDE, SPIC20CAPK, SPVILRKL and WEKAFKI.

